# Development and future perspectives of behavioral medicine in Japan

**DOI:** 10.1186/s13030-016-0054-8

**Published:** 2016-02-24

**Authors:** Shinobu Nomura

**Affiliations:** Faculty of Human Sciences, Waseda University, 2-579-15, Mikajima, Tokorozawa, Saitama 359-1192 Japan

**Keywords:** Type A behavior pattern, Behavior modification, the Japanese version of the Textbook of Behavioral Medicine, Core curriculum of Behavioral Medicine

## Abstract

Development and Future Perspectives of Behavioral Medicine in Japan

The study of the “Type A behavior pattern and myocardial infarction” was one of the main themes in the early stage of Behavioral Medicine. After that, behavior modification came to be widely applied to the treatment of various kinds of chronic diseases, and a general concept of Behavioral Medicine was subsequently formed. The Japanese Society of Behavioral Medicine was established in 1992 and is comprised of researchers in the fields of clinical medicine, social medicine, and psycho-behavioral science. Recently, we devised a core curriculum for behavioral science and behavioral medicine and have published a Japanese version of the “Textbook of Behavioral Medicine” in conformity with it. It is a primer that includes all of the basics and clinical applications of Behavioral Medicine and is edited as a manual that can be utilized in clinical practice. We hope this book will contribute to the development of Behavioral Medicine in Japan, to a more healthy life for our people, and to the improvement of the QOL of our patients. In this paper, I discuss the future perspectives from my personal opinion while looking back on the history of Behavioral Medicine in Japan.

## Background

The study of the “Type A behavior pattern and myocardial infarction” was one of the main themes in the early stage of Behavioral Medicine. Although Psychosomatic Medicine was based on an approach based on “character and disease” from a psychoanalytical viewpoint, the type A behavior pattern was recognized as a risk factor for myocardial infarction in the results of a large-scale predictive study [1]. Furthermore, we were substantially impacted by the study of the prevention of a recurrence of myocardial infarction due to behavior modification from type A to type B [2]. Since then, the study of the type A behavior pattern has grown to include the higher-risk factors “anger and hostility”, but it was the beginning whereby behavior modification came to be widely applied to the treatment of various kinds of chronic diseases and from which the concepts of today’s behavioral medicine have subsequently been formed. Recently, treatments based on behavior modification have become established in daily clinical practice, as it is recognized that behavior modification is essential for the treatment of life-style related diseases and metabolic syndrome.

In this paper, I discuss the future perspectives of Behavioral Medicine from a personal perspective while looking back on the history of Behavioral Medicine in Japan.

## What is Behavioral Medicine?

In the Charter of the International Society of Behavioral Medicine (1990) [3], Behavioral Medicine can be defined as an interdisciplinary field concerned with the development and integration of psychosocial, behavioral, and biomedical knowledge relevant to health and illness and the application of this knowledge to prevention, etiology, diagnosis, treatment and rehabilitation. The scope of “behavioral medicine” extends from research efforts to understanding fundamental biobehavioral mechanisms to clinical diagnosis and intervention to disease prevention and health promotion.

The International Society of Behavioral Medicine was established in 1990 and an international conference has been held every two years since the first international conference was held in Uppsala University, Sweden. Table 1 shows the host countries for each meeting. In Japan, the Japanese Society of Behavioral Medicine was established in 1992 after the 12th International Symposium of the University of Tokyo on Behavioral Sciences [4] was held in 1991.

**Table 1:** Host countries for the meetings of the International Society of Behavioral Medicine

Congress	Year	City (Host country)
1	1990	Uppsala (Sweden)
2	1992	Hamburg (Germany)
3	1994	Amsterdam (Netherlands)
4	1996	Washington D.C. (United States)
5	1998	Copenhagen (Denmark)
6	2000	Brisbane (Australia)
7	2002	Helsinki (Finland)
8	2004	Mainz (Germany)
9	2006	Bangkok (Thailand)
10	2008	Tokyo (Japan)
11	2010	Washington D.C. (United States)
12	2012	Budapest (Hungary)
13	2014	Groningen (Netherland)
14	2016	Melbourne (Australia)

## The history of the International Society of Behavioral Medicine

The Society of Behavioral Medicine was established in the United States in 1979, and interdisciplinary transaction between behavioral science and biomedical science has been subsequently promoted. Because the significance of behavioral medicine in the prevention, diagnosis, and treatment of chronic diseases has been recognized, basic medical research and clinical application into this field have progressed rapidly. In this setting, there were many predictive studies of the “Type A behavior pattern and myocardial infarction” in the 1960s and of the outcome of programs for the prevention of the recurrence of myocardial infarction by behavior modification from Type A to Type B.

After the annual meeting of the Society of Behavioral Medicine was held in 1986, attended by many behavioral scientists and clinical practitioners from many countries of the world, the International Society of Behavioral Medicine was established in 1990 and currently has 26 member societies from around the world. The activity of the International Society of Behavioral Medicine includes scientific conferences, international education seminars, and publishing the “International Journal of Behavioral Medicine”. Prof. Kawakami took the position of president of the International Society of Behavioral Medicine from 2010 to 2012. Many Japanese members have played active roles in many committees. Prof. Tsutsumi is the chair of the scientific program committee for the Melbourne conference in 2016.

## The history of the Japanese Society of Behavioral Medicine

As mentioned above, the 12th International Symposium at the University of Tokyo was an opportunity to stimulate Japanese behavioral scientists. The Japanese Society of Behavioral Medicine started with members integrated into 52 groups from the fields of clinical medicine (psychosomatic medicine), social medicine (public health), and behavioral science (psychology). Its core members were Prof. Ikemi, Prof. Uchiyama, and Prof. Araki. Its activities included an annual meeting, education workshops, and publishing the “Japanese Journal of Behavioral Medicine”. We have continued to carry out unique activities as an interdisciplinary society and to participate actively in international conferences. Many Japanese members participate regularly in international conferences.

In 2008, we hosted the 10th International Conference of Behavioral Medicine, which was highly evaluated for its scientific and management aspects by overseas core members. Table 2 shows the session themes, which included a wide variety of fields from experimental medicine such as psychophysiology and psychoneuroimmunology to clinical medicine and social medicine.

**Table 2:** Outline of the 10th International Congress of Behavioral Medicine in Japan

Date: August 27-30, 2008
Venue: Rissho University, Tokyo
Tracks: Adherence, Aging, Infectious Diseases/SARS/HIV/AIDS, Alcohol/Smoking/ Substance Abuse, Genetics/Environmental Interactions, Cancer, Cardiovascular & Pulmonary Disorders, Childhood & Adolescence, Diabetes/Metabolism/Nutrition /Obesity /Eating disorders, Gender and Health, Health Behaviors, Health Education and Promotion, Health Systems, Policy and Economics, Illness/Illness Affect/Illness Behavior, Illness Behavior, Measurement and Methods, Pain, Musculoskeletal and Neuromuscular Disorders, Physical Activity, Somatoform Disorders/Chronic Fatigue/Medically Unexplained Symptoms, Psychophysiological Disorders & Sleep, Screening & Early Detection, Socioeconomic Factors, Culture & Health, Stress /Psychophysiology/PNI/PNE, Violence/Victimization/PTSD, Work Related Health, Traditional, Integrative & Complementary Medicine

We revised some of the rules of the society in 2009, changing from a group participation system to a personal participation system and accepting many new members. In addition, we planned the activation of research activities by organizing many special interest groups (SIG). The number of members is now about six hundreds.

## Editing of the Japanese version of “the Textbook of Behavioral Medicine”

In 2015, we published a Japanese version of the “Textbook of Behavioral Medicine”, which has been a long-pending objective since the establishment of the Japanese Society of Behavioral Medicine 25 years ago. Recently, behavioral science was included as an important subject for incorporation in our international medical education core curriculum. Also, the importance of behavioral medicine in clinic practice is increasing. Although many European textbooks have been published, there was no Japanese textbook on behavioral medicine. Therefore, we drew up a model core curriculum for behavioral science and behavioral medicine and wrote a textbook in conformity with the model.

As showed in Table 3, this textbook includes extensive contents, including the concepts of behavioral medicine, findings from experimental medicine, understanding the “whole person” from the view-point of psychology and behavioral science, the theory and application of behavioral psychology, and clinical applications to various kinds of chronic disease based on practical methodology.

**Table 3:** Contents of the Japanese version of the “Textbook of Behavioral Medicine”

I. General Remarks
1. What is behavioral medicine? The history and development of behavioral medicine. 2. Biological understanding of behavior (1) Behavior and brain, (2) Neuroendocrinology of behavior, (3) Pharmacology of behavior, (4) Behavior and genetics (epigenetics) 3. Psychological understanding of behavior (1) Information processing of recognition, (2) Behavior and character, (3) Behavior and life cycle, (4) Psychological assessment of behavior 4. Sociological understanding of behavior (1) Interpersonal behavior and social behavior, (2) Behavior and society, (3) Behavioral medicine and biostatistics 5. Holistic understanding of behavior (1) Behavioral science and health science, (2) Behavior and psychosomatic medicine, (3) Behavior and preventive medicine
II. Particular expositions
1. Theories of behavior psychology (1) Learning theory of behavior psychology, (2) Stress coping, (3) Assessment of stress 2. Behavior modification techniques (1) Behavior therapy, (2) Cognitive therapy, (3) Cognitive-behavioral therapy, (4) Mindfulness, (5) Biofeedback, (6) Stage model for behavior modification 3. Application of behavior modification (1) Smoking cessation, (2) Obesity and diabetes, (3) Exercise and physical activity, (4) Cognitive-behavioral therapy for insomnia, (5) Type A behavior, (6) Eating disorders, (7) Depression, (8) Anxiety disorders, (9) Chronic pain, (10) Palliative care, (11) Treatment of alcoholism 4. Communication with patients (1) Medical interview, (2) Motivational interviewing, (3) How to inform bad news, (4) Health behavior and health education (health promotion), (5) What is QOL?

This textbook is a primer that includes all of the “basics and clinical applications of behavioral medicine” and that is written for use in various fields of education, such as medicine, nursing, and psychology, and as a manual that medical staff can utilize in clinical practice. I would like to emphasize that this textbook is very useful for understanding the relations between behavior and health and for treating patients with chronic diseases.

We hope this textbook will contribute to the realization of more healthy lives for our citizens, to the improvement of the quality of life of our patients, and to the development of behavioral medicine.

## Psychosomatic Medicine and Behavioral Medicine

It is well known that psychosomatic medicine developed in the United States in the 1940s, as a psychoanalytical viewpoint toward somatic diseases that made much of the role in psychogenic theory. Since the Biopsychosocial Medical Model was proposed by Engel, GL (1977) [5], psychosomatic medicine has developed in medical research fields related to biology, psychology, and sociology. The basis of Japanese psychosomatic medicine was built by leaders such as Prof. Ikemi and includes as its keywords such concepts as mind-body philosophy, the correlation between body and mind, and holistic medicine. Recently, it has come to include research fields such as mind-brain-body medicine according to the development of brain function studies.

On a memorable occasion, Prof. Ikemi, one of the founders of the Japanese Society of Behavioral Medicine as mentioned above, excitedly told me “psychosomatic medicine and behavioral medicine are important pairs”. He had high expectations for the development of behavioral medicine in Japan. Japanese research into psychosomatic medicine is closely related to behavioral medicine. However, behavioral medicine is characterized as an interdisciplinary science field that includes social medicine and psycho-behavioral science in addition to clinical medicine as psychosomatic medicine.

## Future perspectives

The Japanese Society of Behavioral Medicine is comprised of researchers in the fields of clinical medicine, social medicine, and psycho-behavioral science and performs interdisciplinary research activities. Our purpose is to contribute to the realization of a healthy society by investigating the associations between behavior and disease, the treatment and prevention of various chronic diseases, and the promotion of health. We intend to have scientific evidence on behavioral medicine included in the national health promotion policy known as Health Japan 21.

As mentioned above, the Japanese Society of Behavioral Medicine is one of the main members of the International Society of Behavioral Medicine and our members perform international committee activities on education and research. We would like to encourage the participation of our Japanese Society of Behavioral Medicine members in worldwide scientific activity, especially by our young researchers. In the 21st century, which has been called “the times of stress” and “the times of the heart”, we are convinced that Psychosomatic Medicine and Behavioral Medicine continue to be important research fields that contribute to the welfare and health of the nation as “an important pair”. Although Psychosomatic Medicine has made many great contributions to clinical practice for patients with various psychosomatic diseases and stress-related diseases, Behavioral Medicine has the possibility to expand its findings to welfare worldwide. I hope the research activities of the association members into environmental factors and human health will be extensively promoted in the near future.

## Conclusions

I discussed the history and future perspectives of Behavioral Medicne in Japan. The Japanese Society of Behavioral Medicine was established in 1992 and is comprised of researchers in the fields of clinical medicine, social medicine, and psycho-behavioral science. As Japanese research into psychosomatic medicine is cosely related to behavioral medicne, both are important pairs. I hope the research activities of behavioral medicine will contribute to human health and welfare.
